# Exploring Core Symptoms and Symptom Clusters Among Older Adults with Hypertension–Diabetes Comorbidity: A Network Analysis

**DOI:** 10.3390/healthcare14121646

**Published:** 2026-06-10

**Authors:** Xin Deng, Zhihong Ni, Xinxin Wang, Jiani Hu

**Affiliations:** School of Medicine, Shihezi University, Shihezi 832000, China; dengxin@stu.shzu.edu.cn (X.D.); wangxinxin@stu.shzu.edu.cn (X.W.); hujiani@stu.shzu.edu.cn (J.H.)

**Keywords:** hypertension–diabetes comorbidity, symptom cluster, core symptom, network analysis, older adults

## Abstract

**Highlights:**

**What are the main findings?**

- This study identified three symptom clusters and core symptoms among hypertension–diabetes comorbidity (HDC) patients and explored the interrelationships between these symptoms.
- Fatigue is the most common and severe symptom in HDC patients, which is a core symptom.
- Three main symptom clusters coexist in older adults with HDC: endocrine-neurological disorder symptom cluster, endocrine-metabolic disorder symptom cluster, and general physical symptom cluster.

**What are the implications of the main findings?**

- These findings provide an evidence-based framework for symptom management in older adults with HDC, enabling clinical and community nurses to implement systematic, cluster-based care instead of scattered symptom intervention, improving the pertinence and efficiency of symptom relief.
- Recognizing fatigue as the most common and severe core symptom emphasizes the need to prioritize fatigue screening and intervention. Clinical practice should integrate fatigue assessment into routine follow-up and develop individualized measures to alleviate fatigue and enhance patients’ quality of life.

**Abstract:**

**Background:** Hypertension and diabetes often coexist in older adults, causing complex overlapping symptoms. However, systematic research on their symptom clusters and core symptoms is insufficient, necessitating this study. **Objective**: To explore the composition characteristics of symptom clusters in older adults with HDC, construct symptom networks and identify core symptoms, so as to provide a reference for the development of symptom management programs. **Design**: A cross-sectional study. **Setting:** Patients were continuously recruited from community populations in northern Xinjiang, China, between June 2024 and September 2024. **Participants**: 766 hypertension–diabetes comorbidity patients aged 60 years and older. **Methods:** The symptoms were evaluated using a general information questionnaire and the memory symptom assessment scale (MSAS). The symptom clusters were extracted by systematic cluster analysis, and the symptom network was constructed by R (version 4.4.3) to analyze the central indicators. **Results:** The most common symptom was fatigue (73.5%), followed by dry mouth (64.9%), pain (55.8%), and dizziness (47.5%). Regarding the burden level of symptoms, fatigue was the most burdensome symptom, followed by pain, dry mouth, fatigue, and numbness/tingling in hands/feet. Three clusters were identified: circulatory metabolic disorder symptom cluster, endocrine neurological disorder symptom cluster, and general physical symptom cluster. Network analysis showed that fatigue was the core symptom of the population. **Conclusions:** Community nursing staff should focus on the symptom changes in older adults with HDC, implement precise interventions based on their symptom clusters and core symptoms, and develop personalized symptom management plans to improve the quality of life for patients.

## 1. Introduction

With the accelerating global demographic shift toward aging, non-communicable diseases (NCDs) have emerged as a core public health threat to healthy longevity in older adults. Characterized by insidious onset, multifactorial etiology, and prolonged duration, NCDs frequently lead to the widespread phenomenon of comorbidity, which has become increasingly prominent. According to the World Health Organization, over half (51.0%) of adults aged 60 years or older globally live with multimorbidity, while the prevalence in China’s older adult population stands at 44.9%, rising steeply with advancing age [1,2]. Among these, hypertension and diabetes constitute a common comorbid dyad, termed hypertension–diabetes comorbidity (HDC) [3], whose synergistic pathogenic mechanisms profoundly influence the progression of target-organ damage and functional prognosis. Hypertension exacerbates the progression of diabetic nephropathy, retinopathy, and neuropathy by activating oxidative stress and exacerbating chronic inflammation. Conversely, diabetes further elevates blood pressure through insulin resistance, thereby aggravating atherosclerosis and target-organ damage in the heart, brain, and kidneys associated with hypertension [4]. Global data show that the prevalence of hypertension among individuals with diabetes ranges from 38.5% to 80%, exhibiting significant regional heterogeneity [5]. In China, this prevalence reaches 59.9%, and rises to 54.8% among patients aged 65 years or older [6], highlighting the urgency and uniqueness of HDC as a key prevention and control target in an aging society.

The clinical presentation of HDC is far from a linear summation of typical symptoms of the two diseases, such as dizziness in hypertension or polydipsia and polyuria in diabetes. Older adults with HDC commonly experience cross-system, multidimensional symptoms including fatigue, sleep disturbances, constipation, distal paresthesia or numbness, and depressed mood, and these symptoms frequently occur synchronously, reinforcing one another and evolving dynamically over time [7]. Kim et al. [8] defined such clinically recurrent and pathophysiologically interrelated symptom constellations as symptom clusters. This concept not only provides a theoretical framework for elucidating the synergistic mechanisms underlying multisystem symptoms in comorbidities but also establishes new pathways for precise assessment and personalized interventions in complex comorbid conditions. Within symptom clusters, symptoms interact synergistically through the insulin resistance–vascular damage–inflammatory response axis, exerting more profound impacts than isolated symptoms. This interaction may trigger novel symptoms or severe complications, significantly exacerbating symptom burden in HDC patients. Consequently, it elevates risks of adverse health outcomes, substantially impairs quality of life, and complicates clinical management [9,10,11].

Studies [12] indicate that core symptoms play a pivotal role within symptom clusters, exerting influence over other associated symptoms. Targeted interventions focusing on these core symptoms have been shown to effectively alleviate overall symptom burden in patients. However, most existing research has primarily focused on cross-sectional surveys of individual diseases, such as hypertension or diabetes [13,14], with relatively limited attention given to symptom clusters and core symptom identification in HDC patients.

To systematically reveal the organizational patterns and intrinsic associations of symptoms in older adults with HDC, this study employed an integrated strategy combining hierarchical cluster analysis (HCA) and symptom network analysis to identify symptom clusters and core symptoms.

To systematically explore the organizational patterns and intrinsic correlations of symptoms among older adults with hypertension–diabetes comorbidity, this study adopted hierarchical cluster analysis (HCA) and symptom network analysis to identify symptom clusters and core symptoms.

HCA is a well-established method for exploring the intrinsic structure of symptoms. It does not require pre-specification of the number of symptom clusters and constructs a dendrogram by calculating the co-occurrence similarity among symptoms. This approach objectively identifies the intrinsic clustering structure of symptoms and clearly reveals the macroscopic grouping features and underlying organizational principles of symptom clusters. HCA has been widely applied in the symptom analysis of populations with chronic disease [15]. Symptom network analysis, based on the Gaussian graphical model (GGM), constructs a partial correlation network to accurately estimate the strength of direct association between any two symptoms, controlling for the influence of all other symptoms [16]. In the network, nodes represent symptoms and edges represent statistically significant partial correlations after multiple testing correction. Edge weight reflects the magnitude of the association strength. By calculating the strength centrality of each node, which is defined as the sum of the absolute partial correlation strengths between that symptom and all other symptoms, this method robustly identifies core symptoms that serve as the most pivotal hubs within the network [17]. This approach overcomes the key limitation of conventional correlation analysis, which cannot distinguish direct associations from indirect associations, thereby providing critical insights into potential causal pathways among symptoms [18].

Existing studies have applied cluster analysis or network analysis to investigate symptom characteristics in patients with chronic diseases. However, most of these studies have focused on single disease populations, such as those with simple hypertension or type 2 diabetes, as well as cancer patients. In contrast, research specifically addressing older adults with HDC remains limited. Moreover, few studies have jointly applied hierarchical cluster analysis and symptom network analysis to simultaneously characterize symptom grouping patterns and identify core symptom nodes in this population, whose clinical complexity is further compounded by age-related physiological declines and polypharmacy. In summary, this study aimed to (1) use hierarchical cluster analysis to identify symptom clusters in older adults with HDC; and (2) apply network analysis to explore symptom interrelationships and determine core symptoms. The two methods were integrated synergistically: cluster analysis delineated the macro-level organization of symptom groups, whereas network analysis elucidated the direct pairwise associations and centrality metrics of individual symptoms. These findings are intended to provide essential theoretical foundations and practical pathways for developing digital health intervention tools that target the symptom network such as artificial intelligence-driven early warning systems and personalized symptom management strategies.

## 2. Methods

The study was designed and reported in accordance with the Strengthening the Reporting of Observational Studies in Epidemiology (STROBE) statement for cross-sectional studies [19].

### 2.1. Design

A cross-sectional study.

### 2.2. Study Participants and Setting

This study enrolled HDC patients registered in Shihezi City, Xinjiang, from June to September 2024. Inclusion criteria were as follows: ① permanent residents (residing duration ≥ 6 months); ② age ≥ 60 years; ③ diagnosis of hypertension based on existing literature [20] and diagnosis of type 2 diabetes based on the Chinese Type 2 Diabetes Prevention and Treatment Guidelines (2020 Edition) [7], with both conditions confirmed; ④ clear consciousness and ability to complete the questionnaire independently; ⑤ voluntary participation with written informed consent obtained prior to study initiation. Exclusion criteria included: ① mental disorders or cognitive impairment; ② severe organ dysfunction (e.g., cardiac, cerebrovascular, or renal failure); ③ malignant tumors or life-threatening conditions; and ④ concurrent participation in other clinical trials.

### 2.3. Sample

For sparse networks consisting of 20 or fewer nodes, a sample size of at least 350 is recommended to achieve moderate sensitivity, high specificity, and high edge weight correlation [21]. In this study, the network consisted of twelve nodes, and the final sample included 766 participants, which was considered adequate for data analysis.

### 2.4. Measurements

#### 2.4.1. General Information Questionnaire

Designed by the researchers based on the existing literature [20] including demographic characteristics (gender, age, educational attainment, marital status, medical expense payment method, family residence, and average monthly household income), disease-related characteristics (duration of illness and number of complications), and individual characteristics (smoking history and drinking history).

#### 2.4.2. Memorial Symptom Assessment Scale (MSAS)

Developed by Portenoy et al. [22], the MSAS assesses physical and psychological symptoms experienced by cancer patients over the preceding week. This scale presents favorable applicability across a variety of chronic non-cancer conditions, including patients with heart failure, chronic obstructive pulmonary disease, and metabolic-circulatory multimorbidity [23,24]. It comprises 32 symptoms, with distinct evaluation methods: the first 24 symptoms, rated on frequency, severity, and distress; and the last 8 symptoms, rated on severity and distress. The frequency and severity were rated on a 1–4 Likert scale, while the distress level was rated on a 0–4 Likert scale. If no symptoms are present, the symptom score is 0; if the symptom is present, the scores for the first 24 symptoms are the average of the frequency, severity, and distress scores, and the scores for the last 8 symptoms are the average of the severity and distress scores. The higher the symptom score, the more severe the symptom experience and the greater the associated distress. No fixed clinical cutoff score is established for the total score of the MSAS. The Chinese version adapted by Chang [25] yielded a Cronbach’s α coefficient ranging from 0.79 to 0.87, with a validity coefficient of 0.94. In this study, the Cronbach’s α = 0.830, KMO = 0.843, and Bartlett’s sphericity test *p* < 0.001, confirming robust reliability and suitable factor structure.

### 2.5. Data Collection and Quality Control

Prior to the collection, formal approval was obtained from relevant departments of the urban community health service center. Data were collected from June 2024 to September 2024. A registry list of older adults with HDC was retrieved from community health records, with study participants selected via simple random sampling. Each eligible individual on the registry was assigned a unique identification number. Simple random sampling was then performed using a random-number generator to select participants, ensuring unbiased recruitment from the overall eligible population. Two trained investigators conducted structured household visits to explain the survey purpose, procedures, and required data items. After obtaining written informed consent, participants chose to either self-complete the questionnaire or undergo face-to-face interviewer-administered surveys. All completed questionnaires were checked on-site for logical validity and completeness; any missing items were supplemented and completed immediately on the spot. For data management, paper-based questionnaires were double-entered into Epidata 3.1 by two independent researchers. Consistency checks confirmed data accuracy prior to statistical analysis.

### 2.6. Data Analysis

Data analysis was conducted using SPSS (version 27.0) and R (version 4.4.3). All network construction, centrality calculation, and stability testing followed standard reporting specifications for symptom network analysis. Analytical parameters and model settings were kept consistent to ensure methodological transparency and result reproducibility.

#### 2.6.1. Descriptive Analysis

Prior to analysis, the normality of continuous data was tested using the Shapiro–Wilk test [26]. Continuous variables with normal distribution were summarized as the mean ± standard deviation, while the non-normal distribution was summarized as median and interquartile range. Categorical variables were described using frequencies and percentages.

#### 2.6.2. Cluster Analysis

Hierarchical cluster analysis (HCA) was selected for its ability to identify inherent symptom groupings without requiring predefining cluster numbers, making it suitable for exploring unknown symptom structures in chronic multimorbidity populations. Ward’s minimum variance method was used with squared Euclidean distance to minimize within-cluster variance and maximize between-cluster differences. Only symptoms with a prevalence ≥ 15% were included to ensure clinical meaningfulness and statistical stability [27]. The optimal number of clusters was determined visually from the dendrogram combined with clinical interpretability.

#### 2.6.3. Network Estimation

The qgraph package constructed a symptom severity network based on Spearman correlation matrices regularized by the EBICglasso algorithm (graphical LASSO). In this network, symptoms are represented as nodes, edges between nodes indicate partial correlation strength (thicker edges = stronger correlations), and node positions are optimized using the Fruchterman–Reingold force-directed algorithm, placing highly interconnected nodes centrally. The mgm package estimated node predictability, where symptoms can be predicted by neighboring symptoms.

#### 2.6.4. Centrality Estimation

Three centrality indices were calculated to identify core symptoms:

Strength centrality: the sum of the absolute weights of all edges connected to a node, reflecting the overall connectivity of the symptom.

Closeness centrality: the reciprocal of the total distance from one node to all other nodes; higher values indicate a more central position in the network.

Betweenness centrality: the number of shortest paths passing through a node, reflecting its bridging role in the network.

#### 2.6.5. Accuracy and Stability Estimation

Non-parametric bootstrap (via the bootnet package) estimated 95% confidence intervals for centrality metrics. CS-coefficient > 0.25 indicates acceptable stability, while values above 0.50 reflect good stability of centrality indices [28]. All tests were two-sided with statistical significance at *p* < 0.05.

### 2.7. Institutional Review Board Statement

This study was conducted in accordance with the Declaration of Helsinki and approved by the Institutional Review Board of the First Affiliated Hospital of Shihezi University (Approval No.: KJ2023-506-02; Date: 15 January 2024).

## 3. Results

### 3.1. Characteristics of the Participants

A total of 766 patients were enrolled in this study. The mean age of older adults with HDC was 75.29 ± 7.57 years. The cohort comprised predominantly females (62.1%), while 68.4% were married. Additional demographic characteristics are presented in Table 1.

### 3.2. Symptom Experience of Older Adults with HDC

The symptom profile of older adults with HDC is summarized in Table 2. For clarity, symptoms with a prevalence rate lower than 15% are presented in Appendix A, Table A1. The most prevalent symptoms were fatigue (73.5%), dry mouth (64.9%), pain (55.8%), and dizziness (47.5%). Based on the median severity scores, the most severe manifestations were fatigue, followed by pain [1 (0, 2)], dry mouth [1 (0, 2)], and numbness/tingling in the hands/feet [1 (0, 2)]. Sexual dysfunction was reported less frequently.

### 3.3. Symptom Cluster Analysis

**Figure 1:**
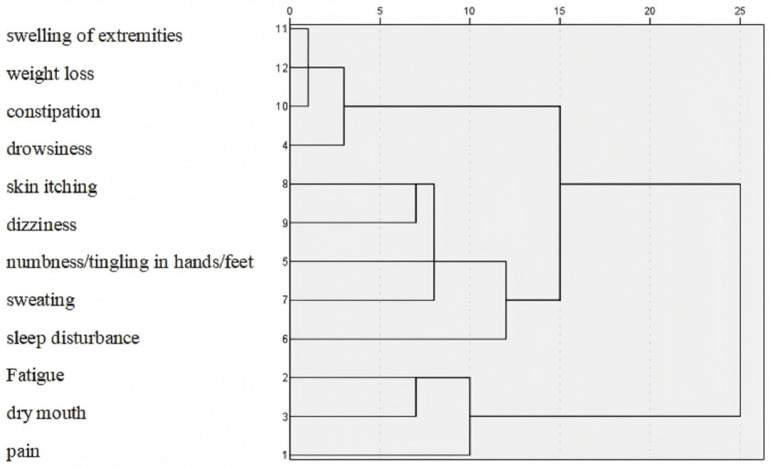
Symptom system clustering spectrum diagram for older adults with HDC.

A hierarchical cluster analysis was conducted on 12 symptoms with an incidence rate ≥ 15%, as illustrated in Figure 1. Three distinct clusters were identified. Based on symptom characteristics and clinical insights, these clusters were designated as follows: the circulatory–metabolic disorder symptom cluster, comprising swelling of extremities, weight loss, constipation, and drowsiness; the endocrine-neurological disorder symptom cluster including skin itching, dizziness, numbness/tingling in hands/feet, sweating, and sleep disturbances; and the general physical symptom cluster, encompassing fatigue, dry mouth, and pain.

### 3.4. Network and Centrality Analysis of Symptom Clusters in Older Adults with HDC

**Figure 2:**
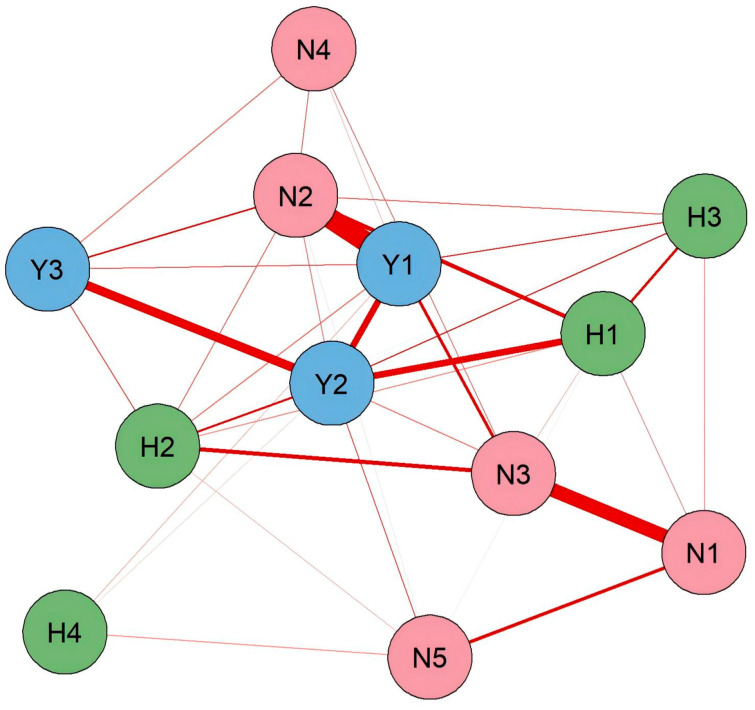
Symptom cluster network analysis of older adults with HDC. Y1: pain. Y2: fatigue. Y3: dry mouth. N1: skin itching. N2: dizziness. N3: numbness/tingling in hands and feet. N4: sweating. N5: sleep disturbance. H1: swelling of extremities. H2: weight loss. H3: constipation. H4: drowsiness.

**Figure 3:**
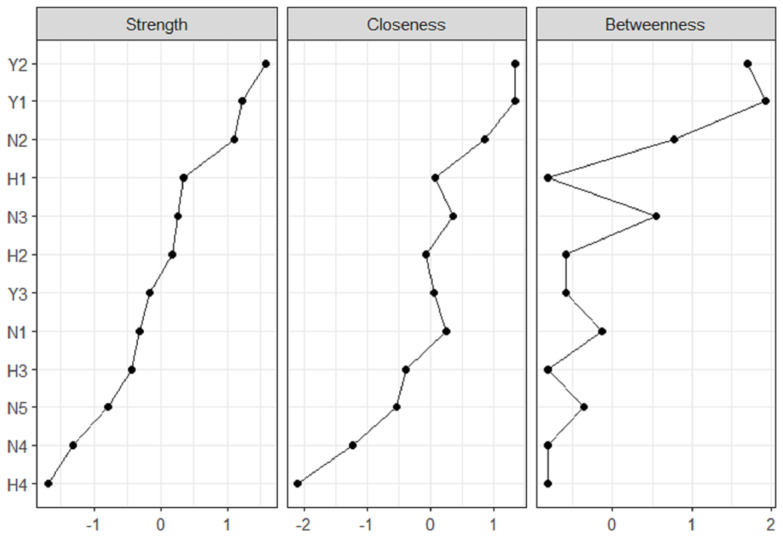
Symptom-centered indicators for older adults with HDC. Y1: pain. Y2: fatigue. Y3: dry mouth. N1: skin itching. N2: dizziness. N3: numbness/tingling in hands and feet. N4: sweating. N5: sleep disturbance. H1: swelling of extremities. H2: weight loss. H3: constipation. H4: drowsiness.

Based on the symptom clustering results obtained from the hierarchical cluster analysis, an undirected weighted symptom network model was constructed for the above 12 symptoms to further analyze the correlation intensity between symptoms and the characteristics of core symptoms, with the results shown in Figure 2 and Figure 3. The symptom cluster network for older adults with HDC identified the top three most strongly correlated symptom pairs based on edge thickness: numbness/tingling and pain in hands/feet, fatigue and dry mouth, and drowsiness and sleep disturbances. Centrality analysis demonstrated fatigue as dominant in strength centrality (rs = 0.92), followed by pain (rs = 0.84), and numbness/tingling in hands/feet (rs = 0.81). Pain exhibited the highest betweenness centrality (rb = 12), surpassing fatigue (rb = 11) and numbness/tingling in hands/feet (rb = 7), while fatigue and pain shared peak closeness centrality values (rc = 0.007 and rc = 0.007, respectively), with numbness/tingling in hands/feet at rc = 0.005 (Figure 3). Stability testing confirmed robust coefficients for strength (0.672) and closeness centrality (0.517), with betweenness centrality (0.439) demonstrating acceptable stability. According to standard criteria, strength and closeness centrality presented good stability, whereas betweenness centrality remained within the acceptable stability range.

## 4. Discussion

### 4.1. The General Physical Symptom Cluster

The general physical symptom cluster constituted the core symptom group among older adults with HDC and should be a priority in clinical management. The results of the hierarchical cluster analysis indicate that it encompassed three key symptoms: fatigue, dry mouth, and pain—all of which are the most prevalent and burdensome symptoms in the study population, as clearly reflected in Table 2. This cluster was defined as the core symptom cluster in older adults with HDC due to fatigue exhibiting the highest strength centrality and betweenness centrality, combined with pain demonstrating maximal closeness centrality. Its pathogenesis involves multisystem interactions [29]: diabetes-induced impairment of glucose utilization, electrolyte loss, and oxidative stress lead to fatigue; hyperglycemia-induced osmotic diuresis and salivary gland damage cause dry mouth; while neuropathy and vascular ischemia result in pain. Concurrently, hypertension-induced increased cardiac load, microcirculatory disturbances, and diuretic use are closely correlated with higher severity of fatigue and dry mouth and intensify ischemic pain by accelerating vascular damage [30,31]. These mechanisms indicate that community healthcare providers should conduct comprehensive clinical assessments of older adults with HDC, focusing specifically on the severity of dry mouth, the level of fatigue, and changes in pain. Integrated intervention strategies should be adopted, including symptomatic support (e.g., potassium supplementation, improvement of microcirculation), optimization of medication regimens (avoiding the use of drugs that exacerbate these symptoms), and lifestyle modifications (guiding patients to maintain fluid balance). This approach aims to disrupt multisystem damage interactions and improve patients’ overall health outcomes.

### 4.2. The Endocrine-Neurological Disorder Symptom Cluster

Based on the results of hierarchical cluster analysis, the endocrine-neurological disorder symptom cluster comprised five symptoms: numbness/tingling in hands/feet, skin itching, dizziness, abnormal sweating, and sleep disturbances. Notably, numbness/tingling in hands/feet demonstrated the highest strength centrality within this symptom cluster, consistent with recent symptom network analyses of older adults with diabetes by Liu et al. [32]. The underlying pathogenesis involves synergistic damage from hyperglycemia and hypertension: hyperglycemia promotes advanced glycation end product accumulation and polyol pathway activation, which—combined with hypertension-induced vascular wall thickening and hemodynamic abnormalities—collectively disrupt endocrine regulation. This manifests as amplified fluctuations in blood glucose and blood pressure, heightened insulin resistance, and ultimately leads to neurotrophic vasa nervorum lesions and nerve fiber demyelination [33,34,35]. Within this pathological cascade, peripheral neuropathy primarily presents as numbness/tingling in hands/feet [36]; cutaneous nerve ending involvement causes skin itching; autonomic dysregulation of vascular and sweat gland control results in dizziness and abnormal sweating; while neurotransmitter disturbances (e.g., serotonin and melatonin dysregulation) induce sleep disorders [37,38]. These symptoms interact reciprocally, forming a vicious cycle that exacerbates endocrine-neurological dysfunction. Consequently, community nursing practice should implement stratified interventions: ① regular follow-ups focusing on the frequency, severity, and progression of numbness/tingling symptoms in hands/feet with detailed documentation to support clinical diagnosis; ② enhance skin management including maintaining skin cleanliness/moisture, rigorous foot inspections, and early screening/intervention for complications (e.g., foot ulcers, fungal infections); ③ combined dietary/exercise guidance, and use medication as prescribed by a doctor when necessary; ④ prevention of orthostatic dizziness through gradual positional changes, avoidance of sudden standing, and supervised daily activities; ⑤ metabolic monitoring with regular blood pressure/sugar checks and timely reporting of abnormalities; and ⑥ optimizing the sleep environment by guiding patients to select breathable, soft, and comfortable sleepwear, and implementing relaxation training when indicated to improve sleep quality.

### 4.3. The Circulatory–Metabolic Dysfunction Syndrome Symptom Cluster

Circulatory–metabolic dysfunction syndrome is often overlooked by patients in its early stages, so assessment and management should be strengthened. In the hierarchical cluster analysis graph, the circulatory–metabolic disorder symptom cluster encompasses extremity edema, weight loss, constipation, and drowsiness. In older adults with HDC, hypertension-related venous reflux disturbances synergistically interact with diabetic nephropathy-induced hypoalbuminemia to precipitate limb swelling [39]. Concurrently, insufficient insulin secretion impairs glucose utilization, triggering accelerated catabolism of proteins and fats to meet energy demands, thereby promoting weight reduction [40]. Persistent hyperosmolar states disrupt gastrointestinal motility, manifesting as weakened peristalsis, difficulty in defecation with hardened stool consistency, and consequent constipation [41]. Furthermore, hypertension-driven macrovascular sclerosis/stenosis and diabetes-mediated microvascular injury collectively induce cerebral circulatory impairment, resulting in cerebral hypoxia-ischemia and compromised energy metabolism—a pathophysiological cascade clinically expressed as lethargy, progressive drowsiness, and even somnolence [42]. These manifestations constitute early-warning indicators of circulatory–metabolic dysregulation [43], but initial symptoms are frequently misattributed to physiological aging, leading to clinical neglect. Through symptom exacerbation, structural damage within the circulatory–metabolic system typically progresses, potentially establishing a vicious cycle that delays therapeutic intervention [44]. Consequently, community healthcare practitioners managing this symptom complex should check the comorbidity-risk health records of middle-aged/older adults with HDC and regularly monitor extremity edema severity, body weight trajectory, defecation frequency, and mental status while implementing proactive health education on disease self-monitoring, dietary modification (low-sodium/low-fat, high-quality protein, adequate fiber), moderate aerobic exercise (e.g., walking, tai chi), and the avoidance of prolonged standing/sitting to collectively ameliorate microcirculatory dysfunction.

### 4.4. Fatigue Is the Core Symptom

In the network analysis, fatigue showed the highest strength (rs = 0.92) and closeness centrality and the most extensive interconnections with other symptoms, suggesting its potential role as a key core symptom among older adults with HDC. This positions fatigue as a pivotal candidate for targeted symptom management in this population. These findings are consistent with previous symptom network analyses conducted in older adults with chronic metabolic multimorbidity [45]. Furthermore, robust epidemiological evidence confirms that fatigue is highly prevalent among individuals with diabetes [46], thereby reinforcing its prominent clinical relevance as identified in the present study. Mechanistically, the hypothalamic–pituitary–adrenal axis in these patients participates in stress response and energy storage regulation; neuroendocrine dysregulation disrupts internal homeostasis, thereby inducing fatigue [47]. Chronic fatigue sustains a stress state, which may cause neurotransmitter imbalances and perturbing the sleep–wake rhythm, which triggers sleep disorders [48]. Consequently, for older adults with HDC with multiple concurrent symptoms, fatigue could be considered a potential priority intervention target to diminish its interactive linkages within the symptom network and enhance symptom management efficacy. Interventions must include: gradually increasing physical activity according to the patient’s adaptive capacity to improve fitness and endurance, thereby alleviating fatigue [49]; providing science-based dietary guidance aligned with nutritional status and preferences to encourage the intake of protein, vitamins, and mineral-rich foods, thus ensuring adequate nutrition for functional improvement; addressing psychological stress and negative emotions through counseling, mental health education, and relaxation techniques (e.g., meditation, deep breathing) to build resilience and mitigate psychologically exacerbated fatigue; and proactively implementing health education to disseminate disease knowledge and fatigue management strategies, empowering self-management and holistic health improvement through active patient engagement [50].

Based on the edge thickness and edge weight of the symptom network shown in Figure 2 and Figure 3, the three strongest associations were observed between numbness/tingling in hands and feet and pain, between fatigue and dry mouth, and between drowsiness and sleep disturbance. Notably, fatigue and pain also exhibited a relatively strong association in the network structure. This robust linkage suggests that fatigue and pain frequently co-occur and mutually reinforce each other among older adults with HDC. Persistent physical pain contributes to physical exhaustion and psychological burden, thereby exacerbating fatigue severity [51]. Consequently, the bidirectional relationship between fatigue and pain should be explicitly integrated into early symptom assessment [52]. Clinicians should perform synchronized evaluation and deliver coordinated, symptom-targeted interventions for both fatigue and pain, which supports the implementation of precise, individualized core symptom management in older adults with HDC.

It should be noted that the most common symptoms observed in this study (such as fatigue, pain, dry mouth, etc.) may be associated with long-term medication use in older adults with HDC. Older adults with HDC typically require the long-term use of antihypertensive and antidiabetic medications, several of which may produce corresponding side effects. For instance, certain antihypertensive drugs (such as beta-blockers) may cause fatigue and dizziness, while some antidiabetic agents (such as sulfonylureas) may induce gastrointestinal reactions like constipation [53]. These factors may influence the identification of the symptom cluster.

## 5. Strengths and Limitations

The strength of this study lies in the synergistic application of hierarchical clustering analysis and network analysis. Hierarchical clustering precisely identified the symptom clustering patterns among older adults with HDC, delineating symptom clusters and providing clear grounds for classifying symptom spectra. Network analysis, meanwhile, delved into the interrelationships between symptoms and pinpointed core symptoms, compensating for the limitation of clustering analysis in revealing individual symptom associations. The combined use of these two methods enabled a comprehensive analysis of the symptom characteristics in older adults with HDC, offering targeted support for clinical symptom management.

However, this study adopted a cross-sectional design, which only captures symptom characteristics at a single time point and cannot infer causal relationships among symptoms. Longitudinal research is required to explore the dynamic changing patterns of symptom clusters. Second, all participants were recruited only from northern Xinjiang, without involving populations from other regions. Differences in lifestyle, cultural background, treatment adherence and healthcare resource utilization across regions may affect the manifestation characteristics of symptom clusters and reduce the external validity of the findings. Future multicenter and large-sample studies covering multiple regions are warranted to further explore this issue.

## 6. Conclusions

Older adults with HDC frequently present with multiple concurrent symptoms, which often manifest as symptom clusters. This study employed systematic clustering analysis to identify symptom clusters and constructed a symptom network using network analysis. Three primary symptom clusters were identified: endocrine-neurological disorder symptom cluster, circulatory–metabolic disorder symptom cluster, and general physical symptom cluster, with fatigue emerging as the core symptom. Clinicians are encouraged to conduct early comprehensive symptom assessments, prioritize fatigue as the key intervention target, and integrate holistic assessment with targeted interventions to improve symptom management and patients’ quality of life. Future research should include multicenter, large-sample studies across diverse geographic regions and incorporate longitudinal follow-up to elucidate the dynamic evolution of symptom clusters.

## Figures and Tables

**Table 1:** Characteristics of the older adults with HDC (*n* = 766).

Characteristics	Categories	*n* (%)
Age (years)	60–74	323 (42.2)
	≥75	443 (57.8)
Sex	Male	290 (37.9)
	Female	476 (62.1)
Education level	Primary school and below	424 (55.4)
	Junior high school	199 (26.0)
	High school, technical secondary school and above	143(18.6)
Marriage	Married	524 (68.4)
	Unmarried	242 (31.6)
Medical payment methods	Medical insurance for urban employees	606 (79.1)
	Medical insurance for urban residents	160 (20.9)
Monthly household income per capita (yuan)	<3000	194 (25.3)
	3000–5000	370 (48.3)
	>5000	202 (26.4)
Duration of hypertension (years)	<5	87 (11.4)
	5–10	122 (15.9)
	>10	557 (72.7)
Duration of diabetes (years)	<5	139 (18.2)
	5–10	145 (18.9)
	>10	482 (63.9)
Comorbidity	Yes	445 (58.1)
	No	321 (41.9)
Smoking	Never smoker	579 (75.6)
	Current smoker	53 (6.9)
	Former smoker	134 (17.4)
Drinking	Never drinker	583 (76.1)
	Current drinker	81 (10.6)
	Former drinker	102 (13.3)

Notes: HDC = hypertension–diabetes comorbidity; *n* = number.

**Table 2:** Symptom experience scores in older adults with HDC (*n* = 766).

Symptoms	Prevalence	Symptom Experience [Scores, Median (P25, P75)]			
	*n* (%)	Intensity	Severity	Distress	Symptom Scores
fatigue	563 (73.5)	3.00 (0, 3.00)	2.00 (0, 2.00)	1.00 (0, 2.00)	2.00 (0, 2.33)
Dry mouth	497 (64.9)	2.00 (0, 4.00)	1.00 (0, 2.00)	1.00 (0, 2.00)	1.33 (0, 2.33)
Pain	435 (55.8)	2.00 (0, 4.00)	1.00 (0, 2.00)	1.00 (0, 2.00)	1.67 (0, 2.67)
Dizziness	363 (47.4)	0 (0, 2.00)	0 (0, 2.00)	0 (0, 1.00)	0 (0, 1.37)
Numbness/tingling in hands/feet	341 (44.5)	0 (0, 3.00)	0 (0, 2.00)	0 (0, 2.00)	0 (0, 2.00)
Sleep disturbances	329 (43.0)	0 (0, 3.00)	0 (0, 2.00)	0 (0, 2.00)	0 (0, 2.33)
Sweats	323 (42.1)	0 (0, 3.00)	0 (0, 1.00)	0 (0, 1)	0 (0, 1.67)
Skin itching	294 (38.4)	0 (0, 2.00)	0 (0, 2.00)	0 (0, 1)	0 (0, 1.67)
Constipation	217 (28.3)	-	0 (0, 1.00)	0 (0, 0)	0 (0, 0.50)
Swelling of extremities	193 (25.2)	-	0 (0, 1.00)	0 (0, 0)	0 (0, 0.50)
Drowsiness	188 (24.5)	0 (0, 0)	0 (0, 0)	0 (0, 0)	0 (0, 0)
Weight loss	127 (16.6)	-	0 (0, 0)	0 (0, 0)	0 (0, 0)

Notes: HDC = hypertension–diabetes comorbidity; *n* = number; P25 = 25th percentile; P75 = 75th percentile.

## Data Availability

The data presented in this study are available upon request from the corresponding author due to privacy restrictions.

## Appendix A

**Table A1:** Symptom experience scores (Prevalence < 15%) in older adults with HDC (*n* = 766).

Symptoms	Prevalence	Symptom Experience [Scores, Median (P25, P75)]			
	*n* (%)	Intensity	Severity	Distress	Symptom Scores
Feeling bloated	81 (10.6)	0 (0, 0)	0 (0, 0)	0 (0, 0)	0 (0, 0)
Lack of appetite	80 (10.4)	0 (0, 0)	0 (0, 0)	0 (0, 0)	0 (0, 0)
Changes in skin	78 (10.2)		0 (0, 0)	0 (0, 0)	0 (0, 0)
cough	74 (9.7)	0 (0, 0)	0 (0, 0)	0 (0, 0)	0 (0, 0)
Hair loss	70 (9.1)		0 (0, 0)	0 (0, 0)	0 (0, 0)
Shortness of breath	58 (7.6)	0 (0, 0)	0 (0, 0)	0 (0, 0)	0 (0, 0)
Diffficulty concentrating	55 (7.2)	0 (0, 0)	0 (0, 0)	0 (0, 0)	0 (0, 0)
Worrying	33 (4.3)	0 (0, 0)	0 (0, 0)	0 (0, 0)	0 (0, 0)
Nausea	28 (3.7)	0 (0, 0)	0 (0, 0)	0 (0, 0)	0 (0, 0)
Feeling irritable	24 (3.1)	0 (0, 0)	0 (0, 0)	0 (0, 0)	0 (0, 0)
Urinary accidents	23 (3.0)	0 (0, 0)	0 (0, 0)	0 (0, 0)	0 (0, 0)
Feeling nervous	23 (3.0)	0 (0, 0)	0 (0, 0)	0 (0, 0)	0 (0, 0)
Diarrhea	22 (2.9)	0 (0, 0)	0 (0, 0)	0 (0, 0)	0 (0, 0)
Mouth sores	17 (2.2)		0 (0, 0)	0 (0, 0)	0 (0, 0)
Diffficulty swallowing	11 (1.4)	0 (0, 0)	0 (0, 0)	0 (0, 0)	0 (0, 0)
Vomiting	9 (1.2)	0 (0, 0)	0 (0, 0)	0 (0, 0)	0 (0, 0)
Change in the way food tastes	8 (1.0)		0 (0, 0)	0 (0, 0)	0 (0, 0)
Feeling sad	1 (0.1)	0 (0, 0)	0 (0, 0)	0 (0, 0)	0 (0, 0)
“I don’t look like myself”	1 (0.1)		0 (0, 0)	0 (0, 0)	0 (0, 0)
Problems with sexual interest or activity	0 (0.0)	0 (0, 0)	0 (0, 0)	0 (0, 0)	0 (0, 0)
